# Outcome after Posterior Vertebral Column Resection in Patients with Severe Osteoporotic Fractures—A Retrospective Analysis from Two Centers

**DOI:** 10.3390/medicina58020277

**Published:** 2022-02-12

**Authors:** Leon-Gordian Koepke, Lukas Weiser, Martin Stangenberg, Marc Dreimann, Annika Heuer, André Strahl, Lennart Viezens

**Affiliations:** 1 Division of Spine Surgery, Department of Trauma and Orthopedic Surgery, University Medical Center Hamburg-Eppendorf, 20246 Hamburg, Germany; m.stangenberg@uke.de (M.S.); m.dreimann@uke.de (M.D.); ann.heuer@uke.de (A.H.); l.viezens@uke.de (L.V.); 2 Clinic for Trauma, Orthopedics and Plastic Surgery, University Medical Center Göttingen, Georg-August-Universität, 37075 Göttingen, Germany; lukas.weiser@med.uni-goettingen.de; 3 Mildred Scheel Cancer Career Center HaTriCS4, University Medical Center Hamburg-Eppendorf, 20246 Hamburg, Germany; 4 Division of Orthopedics, Department of Trauma and Orthopedic Surgery, University Medical Center Hamburg-Eppendorf, 20246 Hamburg, Germany; a.strahl@uke.de

**Keywords:** osteoporotic fractures, spine, spinal fusion, kyphosis, osteoporosis, geriatrics

## Abstract

*Background and Objectives:* In osteoporotic fractures of the spine with resulting kyphosis and threatening compression of neural structures, therapeutic decisions are difficult. The posterior vertebral column resection (pVCR) has been described by different authors as a surgical treatment in a single-stage posterior procedure. The aim of this study is to evaluate midterm outcomes of patients treated by pVCR due to severe osteoporotic fractures. *Materials and Methods:* Retrospective data analysis of all the patients treated for osteoporotic fractures by pVCR from 2012–2020 at two centers was performed. Demographic data, visual analog scale (VAS), Frankel scale (FS), Karnofsky performance status (KPS), radiological result and spinal fusion rates were evaluated. *Results:* A total of 17 patients were included. The mean age was 70 ± 10.2 y. The mean VAS decreased significantly from 7.7 ± 2.8 preoperatively to 3.0 ± 1.6 at last follow-up (*p* < 0.001) and the segmental kyphosis decreased from 29.4 ± 14.1° to 7.9 ± 8.0° (*p* < 0.001). The neurologic function on the FS did not worsen in any and improved in four of the patients. The median KPS remained stable over the whole observation period (70% vs. 70%). Spinal fusion was observed in nine out of nine patients who received CT follow-up >120 days after index surgery. *Conclusions:* This study showed that pVCR is a safe surgical technique with few surgical complications and no neurological deterioration considering the cohort. The patients’ segmental kyphosis and VAS improved significantly, while the KPS remained stable.

## 1. Introduction

Osteoporotic fractures are a major concern for both individual patients and the entire healthcare system [1,2]. Due to demographic aging, this diagnosis will continue to increase significantly in the coming years (y) [3].

The clinical manifestation and the therapy of osteoporotic fractures of the spine vary. Most of the osteoporotic fractures of the spine can and should be treated conservatively or in a minimally invasive manner. Conservative therapeutic strategies include acute fracture treatment with analgesia, temporary bed rest or immobilization in a frame corset, as well as mandatory secondary prophylaxis through antiresorptive or osteoanabolic medication [4,5,6]. Surgical treatment options range from minimally invasive cementoplasties [7,8,9] through percutaneous instrumentation to open surgical procedures with dorsal and ventral approaches and associated significant surgical trauma [10,11,12].

Osteoporotic fractures are significantly different from traumatic fractures in healthy bone, and the classifications for traumatic injuries cannot be applied to osteoporotic fractures [13]. Therefore, osteoporotic fractures should be classified differently from traumatic fractures and a classification for osteoporotic fractures of the spine (OF classification) was published [13]. According to this classification, there are five degrees of severity, in the range of OF 1–5. While OF 1 and 2 fractures describe stable fractures without a burst component, OF 3–5 fractures are incomplete or complete burst fractures with involvement of the posterior edge and resulting instability. In particular, when these fractures occur in the upper to the middle thoracic spine, hyperkyphosis with sagittal imbalance and stenosis of the spinal canal including impending or manifest myelopathy and consecutive neurological deficits may occur [14,15,16].

These fractures represent a major challenge, as surgical intervention is required to decompress the spinal canal and the sagittal profile needs to be restored to prevent implant failure and adjacent level fractures. At the same time, these patients are often multimorbid and have a significantly increased perioperative risk. Procedures demanding dorsal and ventral approaches to regain adequate decompression and stabilization of the spine might not be suitable for these patients. In the past, posterior vertebral column resection (pVCR) has been described as a possible surgical therapy concept for these patients [14,15,17,18].

Little is known about the mid-and long-term outcome in these patients. Therefore, this study was designed to examine the clinical and radiological midterm outcome after pVCR in daily clinical practice at two tertiary referral centers.

## 2. Materials and Methods

This study is reported according to the Strengthening The Reporting of Observational studies in Epidemiology (STROBE) guideline [19] and was approved by the ethics committee of the Hamburg medical association (WF-053/21). Given the anonymization of the data, no further consultation by the ethics committee and no informed consent were necessary.

The data of all patients treated by pVCR at two tertiary referral centers in the period from 2012 to 2020 were analyzed retrospectively. A total of 110 patients could be identified. In this cohort, 17 patients who underwent 360° decompression and pVCR surgery for a high-grade osteoporotic spine fracture could be identified and were included in the study. A fracture was osteoporotic if the patient had already been diagnosed with osteoporosis by DXA (T-score < −2.5 standard deviations) according to guidelines [20] and there was no other cause for the occurrence of the current fracture. In patients who were not pre-diagnosed with osteoporosis, the fracture was of osteoporotic origin if there was no adequate trauma, an underlying malignant disease was excluded, and the histological specimen obtained intraoperatively confirmed osteoporosis due course.

**Figure 1:**
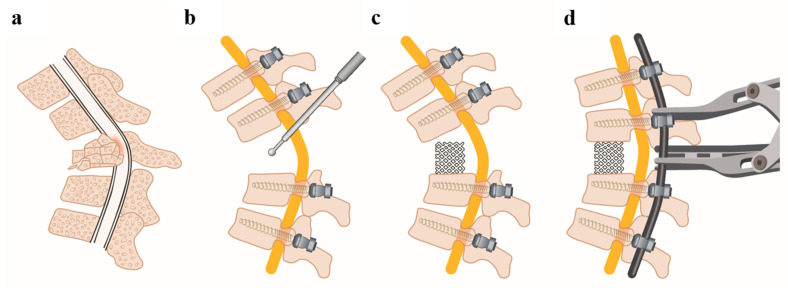
Key steps of the operative technique. (**a**) Scheme of a high-grade osteoporotic spinal fracture with consecutive kyphosis and spinal canal stenosis. (**b**) The fractured vertebral body was surgically removed in a posterior-only procedure (**c**) Harms meshes are inserted in the created cavity as a replacement of the resected vertebral body. (**d**) Using the Harms meshes as a hypomochlion, successive compression and shortening of the posterior spinal elements and, herewith, the lordosation maneuver is carried out.

The surgical procedure of performing pVCR with 360° decompression and osteosynthesis has already been described and published in the past [15,17,21]. Figure 1 schematically shows key steps of the operative technique.

The patient was prone positioned. Open dorsal access to the spine was performed with subperiosteal dissection of the paravertebral muscles. This was followed by the placement of pedicle screws at least two vertebral bodies below and above the fracture (Figure 1b). Subsequently, a temporary rod was inserted unilaterally and fixed before the start of decompression of the spinal canal to protect the neural structures from compression or distortion. This was followed by a careful 360° decompression with resection of both pedicles, the posterior longitudinal ligament and both adjacent discs (Figure 1c). If necessary, between T 3–12, a rhizotomy was performed. This was followed by the fluoroscopic measurement of the height required for the vertebral body replacement to be inserted. Subsequently, the Harms meshes can be inserted into the previously established cavity from both sides (Figure 1c). This was followed by trimming, anatomical prebending, and insertion of the longitudinal rod. The caudal screws were firmly fixed to the rod followed by successive compression and shortening of the posterior spinal elements and herewith lordosation maneuver under fluoroscopic control (Figure 1d). Care should be taken to adhere to the one-third rule [22]. A reduction of one-third is described as safe, a reduction of up to two-thirds is in a warning range, and a reduction of more than two-thirds is in a dangerous range [22]. If this rule is followed and a shortening of more than two-thirds is avoided; in the authors’ experience the use of intraoperative neuromonitoring can be waived. After successful correction of the local kyphosis to physiological values and fixation of all screws to the rods, a spondylodesis was established over the entire height by attaching autologous cancellous bone, which was previously obtained during decompression.

The following clinical parameters were collected and evaluated. Age and sex of the patients were recorded. The patients’ general condition and activity level were assessed using the Karnofsky performance status (KPS). A 10-point visual analog scale (VAS) was used to record and evaluate the pain symptoms of the patients [23]. The preoperative VAS was raised after the initial administration of analgetic medication. The evaluation of the patients’ neurological functions was performed using the Frankel scale (FS) [24]. In addition, general and surgical perioperative complications and revision surgeries were recorded and evaluated.

Preoperative and all postoperative imaging, consisting of conventional radiography (CR) and computed tomography (CT), were independently evaluated by the first and last authors to assess the radiologic outcome of patients. Questionable cases were reevaluated by author M.S. In the preoperative, postoperative, and final follow-up imaging, kyphosis was measured between the cover plate of the adjacent cranial vertebral body and base plate of the caudal adjacent vertebral body. In addition, the height of the cranial and caudal adjacent vertebral body was measured from cover plate to base plate and the height of the cranial and caudal intervertebral disc, in the area of the trailing edge. These heights were averaged to estimate the original height of the fractured and deformed vertebral body. Furthermore, the height between the base plate of the cranial vertebral body and the cover plate of the caudal vertebral body was measured to determine the height of the vertebral body replacement. The percentage of shortening of the spine in the affected area could be calculated from these values. The presence of bony fusion was assumed if there was radiographically clear bony bridging over the resected vertebra in a CT scan > 120 d postoperatively.

For statistical analysis, the software SigmaPlot 13 of Systat Software Inc., San Jose, CA, USA was used. The analysis of the patient data was descriptive. Continuous variables are expressed as mean ± standard deviation. Exceptions are found in the values of KPS and follow-up time. These values are given in median, range, and quartiles (Q_1_/Q_3_). Categorial variables are expressed as number and percentage. The Shapiro–Wilk test was used to test normal distribution. To evaluate the statistically significant differences between the preoperative and follow-up measurement time point, t-test for dependent samples in the case of a normal distribution or the Wilcoxon rank sum test in the case of a non-normal distribution was used. The significance level was *p* < 0.05.

## 3. Results

Parts of the currently analyzed patient collective has already been retrospectively analyzed in the past and the obtained data have been published in a single surgeon study with 10 included patients [15].

### 3.1. Demographic Data and Extent of Surgery

In total, 17 patients were included in the present study; 10 of these patients were female and 7 patients were male (Table 1). The mean age was 71 ± 10.2 y and ranged from 40 y to 83 y (Table 1). The patients were operated by five different experienced spine surgeons at two different centers. The mean operative time was 287 ± 108 min and the postoperative length of stay was 21 ± 15.5 d. Resection of a single vertebral body was performed in 16 cases (94.1%). In one patient (5.9%), the vertebral bodies of two adjacent levels were resected (Table 1). In nine cases (52.9%), a rhizotomy was performed at the level of the resected vertebral body, and in 14 cases (82.4%), cement augmentation of the inserted pedicle screws was performed (Table 1). The mean intraoperative blood loss was 2117.2 mL ± 1431.8 mL.

### 3.2. Clinical Outcome

The retrospective data collection took place in January 2021. The median follow-up was 459 d (range 20–2804; Q1/Q3 66/1033). Regarding preoperative neurological function, 10 of the patients presented an E (58.8%) and 7 patients (41.2%) presented a D on the FS (Table 2). In 13 patients (76.5%), the FS was stable from preoperative to postoperative, and 4 patients (23.5%) showed an improvement from D to E (Table 2). At the time of the last follow-up, an improvement of the FS from D to E occurred in another case (Table 2). Preoperatively there was a mean VAS of 7.7 ± 2.8 and postoperatively a mean of 4.3 ± 1.8. At the time of the last follow-up, the mean VAS has further decreased to 3.0 ± 1.6 (t = 6.15, *p* < 0.001) (Table 2). The median KPS of the patients was 70 (range 50–90; Q_1_/Q_3_ 60/70) preoperatively and remained stable at this level until the last follow-up with no statistical difference (W = 8.0, *p* = 0.164) (Table 2).

Secondary diagnoses, surgical complications, and general complications are shown in Table 3. In four cases (23.5%), a surgical revision due to implant loosening was necessary over time. One of these implant loosenings occurred in the context of a proximal junctional kyphosis (PJK). There was no need for surgical revisions due to neurological impairment or misplacement of implants. In three cases (17.7%) a surgical revision due to a deep wound infection was needed. We know of only one death in the current study collective, this patient died due course, of a cerebral infarction following an occlusion of the arteria cerebri media by a cardiogenic embolus due to a known tachyarrhythmia absoluta.

### 3.3. Radiological Outcome

The values of the radiological outcome are shown in Table 4. Preoperatively, the affected segments showed a mean kyphosis of 29.4° ± 14.1° This was reduced to 7.9° ± 8.0° (W = 91.0, *p* < 0.001) by surgery. The last follow-up showed a mean kyphosis of 10.0° ± 9.6° (W = 91.0, *p* = 0.002). In addition, a mean shortening of 53.5% ± 15.3 was shown.

**Figure 2:**
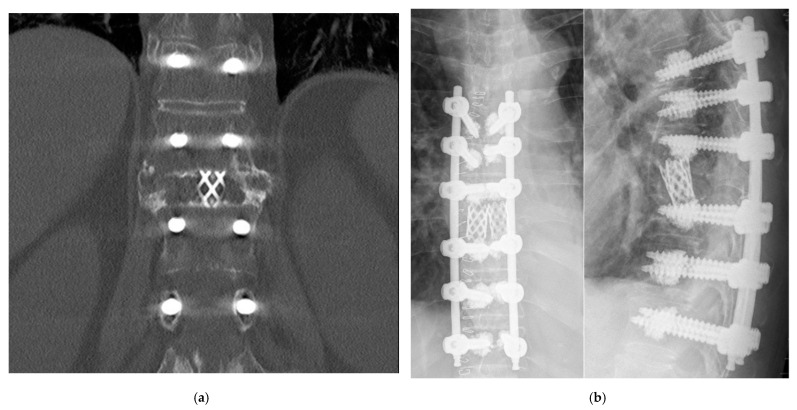
(**a**) Solid bony fusion. This figure shows the typical solid bony fusion between the cranial and caudal adjacent vertebral body, bridging the harms mesh. Usually, the bony fusion occurs bilaterally in a bracketlike manner. (**b**) Typical conventional X-ray after posterior only vertebral column resection in anteroposterior (left) and lateral plane (right): Augmented pedicle screws were inserted three levels above and below the target vertebra. The target vertebra was resected in a posterior-only approach. Harms meshes were inserted. The spine was relordosed and the affected segment compressed. Spondylodesis was established using autologous bone.

Nine of the patients were followed-up by CT > 120 d postoperatively. In all of these patients a solid bony fusion (Figure 2a) could be detected (*n* = 9). Figure 2b shows a typical example of a postoperative CR in a case of an OF 5 fracture of T 9 treated with pVCR.

## 4. Discussion

While most patients with osteoporotic fractures of the spine can be treated conservatively or minimally invasively, a small proportion of patients require more invasive surgical therapy to treat or prevent complications such as hyperkyphosis with resulting pain, immobilization, dyspnea, and neurologic dysfunction. The primary therapeutic goal is to maintain or restore the patient’s ability to walk. To treat patients with collapsed vertebral bodies due to osteoporotic fractures leading to hyperkyphosis and concomitant stenosis of the spinal canal, extensive surgical procedures seem to be inevitable.

In the past, data were presented on 315 patients who underwent instrumented spinal fusion including autologous cancellous bone grafting for an osteoporotic fracture in the thoracolumbar transition with associated neurologic dysfunction of the lower extremities [10]. Operative procedures used included combined vertebroplasty and dorsal instrumentation, anterior spinal fusions (ASF), posterior spinal fusions (PSF), combined anterior and posterior spinal fusions (APSF), posterior three-column osteotomies (3CO), and pVCR. The colleagues concluded that all of these procedures are treatment options with good neurologic outcomes and similar complication rates. However, ASF and PSF are limited in severe osteoporosis, compression of neural structures, and kyphotic deformity. APSF seems to be superior to ASF or PSF in these patients but is associated with higher perioperative risks, especially for the elderly [10,25,26].

Overall, highly invasive surgery appears to be inevitable in patients with advanced osteoporosis, poor bone status, and severe spinal osteoporotic fractures with consecutive kyphotic deformity and concomitant neurologic deficits. Especially in the presence of bony stenosis of the spinal canal, an initial dorsal decompression seems to be necessary. Subsequently, a release must be performed from the anterior side, since a sufficient reduction in the deformity cannot be achieved from the dorsal side due to the weak anchorage of screws in the osteoporotic bone [27,28]. Consequently, another dorsal intervention is necessary to achieve adequate relordosation and establish the spondylodesis. Frequent intraoperative repositioning of the patient or multistage treatment is necessary to perform this dorso-ventrodorsal operative strategy. These procedures are associated with extreme perioperative risks, especially in the thoracic spine where transthoracic access to the ventral spine is required. In particular, pulmonary complications can frequently occur, and geriatric patients who present with pulmonary pre-existing pulmonary and cardiac conditions are at particular risk [25].

Considering these facts, pVCR seems to be a more favorable surgical therapy option in patients with severe kyphotic deformity and stenosis of the spinal canal due to an osteoporotic fracture. The pVCR appears to combine the advantages of simultaneous release, decompression and stabilization of the ventral and dorsal columns while saving the need for a ventral approach to the spine [18] and thus may be the ideal therapy option for elderly patients [15].

Performing a pVCR has been described in the past as a therapeutic option for kyphotic deformities of the spine of various etiologies, such as tumors, infections, and congenital deformities as well as osteoporotic fractures [16,17,21,29,30]. It has been demonstrated that a 360° decompression and osteosynthesis for osteoporotic fractures with kyphotic deformity and spinal stenosis using pVCR is a safe surgical treatment option, in the hands of a single specially gifted surgeon [15]. With this technique, significant correction of the kyphotic deformity was achieved without worsening the patients’ neurological outcome [15].

In this study, it was shown in a multi surgeon approach, that pVCR is a safe, albeit complex surgical treatment option. It can significantly reduce the kyphotic deformity of the spine (*p* < 0.001) to physiological values [31] and restore sagittal balance. Furthermore, the patients’ pain symptoms can be reduced significantly (*p* < 0.001) while keeping the KPS stable.

With increasing demographic aging, geriatric patients aim for a good quality of life and autonomous mobility. Therefore, the establishment of a safe bony fusion after surgical treatment of severe osteoporotic fractures gains relevance regarding the prevention of implant loosening. Especially in osteoporotic bone, such a fusion is difficult to achieve. No consistent data on bony fusion rates after pVCR in severe osteoporotic fractures are available in the current literature. Bony fusion rates after dorsal instrumentation in osteoporotic fractures of 80.5% to 100% have been reported in the literature [32,33,34]. For the first time, this study was able to demonstrate solid bone fusion in all cases evaluated with CT > 120 d after index surgery (*n* = 9).

The mean blood loss in the current study was 2117 mL. It is known that high intraoperative blood loss is associated with higher perioperative morbidity and mortality [35]. However, in severe osteoporotic fractures with consecutive hyperkyphosis and spinal stenosis, correction with high surgical effort might not be avoided. The mean blood loss after ASF, APSF, PSF, and 3CO was compared in the past, and no significant differences were found [10]. Moreover, our data do not differ from the data reported in the literature on typical mean blood loss in pVCR [16,36].

Overall, pVCR appears to be a surgical procedure with non-negligible complication rates, which are reported in the literature to be 22–40.5% [16,36,37]. While neurologic deterioration is one of the most common complications, there was no neurologic deterioration in the actual study cohort. However, loosening of implants occurred in four cases (23.5%).

In the current literature, similar rates of material-associated complications can be found, for example, 10.7% [36] or 18.7% [10]. In all patients who required revision due to material loosening, no cement augmentation of the screws had taken place during the index surgery. No material loosening occurred in patients with augmented screws. When performing pVCR for osteoporotic fractures, it is essential to follow the rules for increasing the stability of pedicle screws [38], and to augment the inserted screws.

Postoperative wound infections also occurred at a rate of 17.7% as a postoperative surgical complication in the currently studied collective. In two of the cases, iatrogenic injuries to the dura mater had occurred during initial care, and in the third case, there was blood loss of >6000 mL. Both iatrogenic injury to the dura [39] and massive blood loss [40,41] are known to be major risk factors for the development of a wound-healing disorder. Patients with these perioperative complications should be monitored particularly carefully postoperatively.

Moreover, risk factors for the occurrence of postoperative complications after pVCR have been described before [36]. They include preoperative neurological limitations, preoperative kyphosis, and fusion of more than five segments [36]. In the current study, eight of the patients (47.1%) showed one, six of the patients (35.3%) showed two, and three of the patients (17.6%) showed all three of the above risk factors. Overall, the present data and the data published in the current literature show that there is a relevant perioperative risk associated with pVCR. This procedure should be performed only after the strictest indication and by experienced surgeons.

However, in patients presenting with an osteoporotic fracture with severe kyphotic deformity, spinal stenosis, and significant impairment of quality of life as well as the failure of other treatment options, pVCR appears to be a safe surgical option. However, it must be performed in a maximum care center in order to manage all kinds of perioperative complications. Furthermore, regardless of the severity of the osteoporotic vertebral body fracture that has occurred, it is mandatory that patients are referred to the osteologist and treated following the guidelines. The stand-alone therapy of the vertebral body fracture does not appear to be sufficient and secondary prophylaxis should be administered.

The main limitations of the current study result from the retrospective study design and the resulting incomplete follow-up. In any case, the analyzed patient collective is a special collective with a rarely performed surgical treatment. The pVCR was used as an ultima ratio to prevent the patient’s continued dependence on opiates, continued immobilization, and the associated risk of potentially fatal complications. In the current retrospective study, a complete clinical and radiological follow-up could only be achieved in 9 of 17 included patients. To overcome this limitation, more studies must follow to further classify and evaluate the pVCR as a procedure for the treatment of the most severe osteoporotic fractures in specific patients. Conducting studies with a prospective study design in which a complete radiological and clinical follow-up, including the acquisition of Quality Adjusted Life Years (QUALY) to measure the clinical outcome and quantify the results of the intervention, seems necessary. The regular use of this type of surgery in special patients according to strict indications allows these studies to be performed.

## 5. Conclusions

We demonstrated that pVCR is a safely feasible surgical option for patients with impending or existing neurological dysfunction and immobilization due to severe osteoporotic fractures after failure of alternative conservative and surgical treatment options. There were few surgical complications and no neurological deterioration considering the cohort. The patients’ segmental kyphosis and pain symptoms improved significantly, while the KPS remained stable. Due to the increasing number of patients presenting with clinical osteoporotic vertebral fractures and simultaneously increasing demand for quality of life, bony fusion seems to be gaining importance. With this work, we demonstrated bony fusion rates of 100% of cases evaluated with CT > 120 d after index surgery (*n* = 9). Overall, pVCR is a safe treatment option for patients with severe osteoporotic fractures with consecutive kyphosis and spinal stenosis and can be performed by an experienced spine surgeon. However, the indication to perform pVCR should be strict and only in individual cases. The risks and chances of this procedure should be discussed in detail with the patients. The primary therapeutic goal is to maintain or restore the patient’s ability to walk.

## Figures and Tables

**Table 1:** Demographic data and extent of surgery. m—male, f—female, BMI—body mass index, T—thoracic vertebra, L—lumbar vertebra, S—sacral vertebra, y—yes, n—no, X—no value available.

Patient	Age	m/f	BMI	Resected Vertebral Body	Highest Instrumented Vertebra	Lowest Instrumented Vertebra	Rhizotomy y/n	Augmentation of Screws y/n
1	72	f	21.97	T 12	T 10	L 2	n	y
2	72	f	21.56	T 12	T 10	L 2	n	y
3	83	f	24.54	L 1	T 11	L 3	y	y
4	81	f	40.01	T 12	T10	L 2	y	y
5	74	m	22	T 8 and 9	T 6	T 12	y	y
6	66	m	37.07	L 1	T 11	L 3	n	y
7	71	f	19.1	L 1	T 6	OS ilium	n	y
8	80	f	17.86	L 2	T 12	L 4	n	y
9	77	m	29.54	L 1	T 11	L 3	n	y
10	77	m	28.41	T 12	T 10	L 2	y	y
11	57	f	22.22	T 6	T 2	S 1	y	y
12	71	f	23.14	T 3	T 1	T 5	n	y
13	65	m	22.53	T 11	T 9	L 1	y	n
14	71	f	17.99	T 9	T 5	T 12	y	y
15	63	m	39.6	L 1	T 10	L 4	n	n
16	40	m	24.5	T 8	T 5	T 11	y	n
17	69	f	23.4	T 9	T 6	L 2	y	y

**Table 2:** Clinical outcome parameters of the patient population. VAS—visual analogue scale, preop—preoperative, postop—postoperative, X—no value.

Patient	VAS Preop	VAS Postop	VAS Last Follow-Up	Frankel Preop	Frankel Postop	FRANKEL Last Follow-Up	Karnofsky Preop	Karnofsky Last Follow-Up
1	10	X	4	E	E	E	70	90
2	7	3	3	D	E	E	80	80
3	8	4	4	D	D	E	50	70
4	10	8	4	D	D	D	50	50
5	10	5	5	D	E	E	70	70
6	10	6	2	E	E	E	90	90
7	10	5	0	D	E	E	50	70
8	10	6	2	E	E	E	80	90
9	7	5	5	E	E	D	60	60
10	10	4	5	E	E	E	60	60
11	3	4	5	E	E	E	60	60
12	8	3	2	E	E	E	70	90
13	1	3	1	D	D	D	70	70
14	8	3	3	D	E	E	70	70
15	3	0	3	E	E	E	60	80
16	8	5	1	E	E	E	70	70
17	8	4	2	E	E	E	70	90

**Table 3:** Secondary diagnoses, surgical and nonsurgical complications. IDDM—insulin-dependent diabetes mellitus, NIDDM—non-insulin-dependent diabetes mellitus, COPD—chronic obstructive pulmonary disease, C—Clostridium.

Secondary Diagnosis	Number of Occurrences	Surgical Complication	Number of Occurrences	Non-Surgical Complication	Number of Occurrences
Cardiac disease	9	Material loosening	4	C. difficile infection	1
Renal failure	2	Iatrogenic dura leak	2	Cardial decompensation	1
IDDM	2	Wound infection	3	Pneumonia	1
NIDDM	1	Seroma	1	Urinary tract infections	4
Malignancy	3				
Obesity	3				
COPD	1				

**Table 4:** Radiological outcome of the patients in the study population. Preop—preoperative, postop—postoperative, y—yes, n—no, X—no value available, d—days.

Patient	Kyphosis Preop in °	Kyphosis Postop in °	Kyphosis Last Follow-Up in °	Shortening in %	Spinal Fusion y/n
1	28	4	5	72	y
2	12	6	2	71	follow-up < 120 d
3	30	1	1	55	follow-up < 120 d
4	31	1	24	79	y
5	53	21	23	61	follow-up < 120 d
6	21	12	14	59	follow-up < 120 d
7	41	10	9	48	y
8	9	8	8	62	y
9	32	6	11	55	y
10	6	2	2	25	follow-up < 120 d
11	41	6	18	60	y
12	X	7	7	40	follow-up < 120 d
13	X	23	23	29	y
14	X	1	1	57	follow-up < 120 d
15	X	1	−4	36	follow-up < 120 d
16	43	25	26	51	y
17	35	0	0	46	y

## Data Availability

The data presented in this study are available within this article. All the data generated or analyzed during this study are included in this published article. A summary of the data can also be provided upon request.
